# Quality of glycemic control has significant impact on myocardial mechanics in type 1 diabetes mellitus

**DOI:** 10.1038/s41598-022-24619-2

**Published:** 2022-11-23

**Authors:** Máté Hajdu, Maren Oedven Knutsen, Vivien Vértes, Noémi Vorobcsuk-Varga, Gergő Molnár, István Wittmann, Réka Faludi

**Affiliations:** 1 Heart Institute, Medical School, University of Pécs, Ifjúság út 13., Pécs, 7624 Hungary; 2 Medicover Clinic, Munkácsy M. u. 15., Pécs, 7621 Hungary; 3 2nd Department of Internal Medicine and Nephrological Center, Medical School, University of Pécs, Pacsirta u.1., Pécs, 7624 Hungary

**Keywords:** Cardiology, Endocrinology

## Abstract

The potential associations between disease duration, glycemic control, and the echocardiographic markers of the myocardial mechanics were investigated in asymptomatic T1DM patients. Seventy T1DM patients (38.2 ± 11.7 years, 46 female) and 30 healthy volunteers were investigated. Besides the conventional and tissue Doppler measurements, left ventricular global longitudinal (GLS) and circumferential (GCS) strain as well as left and right atrial strain parameters were measured with 2D speckle tracking technique. Median HbA_1c_ level was 7.4 (1.8)%. Even when added age and hypertension to the model, current HbA1c level remained independent predictor of left ventricular GLS (p = 0.002), GCS (p < 0.001), mitral e’ (p = 0.018), tricuspid e’ (p = 0.018) and left (p = 0.039) and right atrial conduit strain (p = 0.047) in multiple linear regression models. Correlations between disease duration and the echocardiographic variables lost their significance in multiple models. In patients with a combination of HbA_1c_ ≤ 7.4% and no hypertension, echocardiographic findings did not differ from those in healthy volunteers. Patients with HbA_1c_ > 7.4% and no hypertension and especially patients with coexisting hypertension and HbA_1c_ > 7.4%, exhibited significantly impaired myocardial mechanics. Quality of glycemic control has a significant impact on myocardial mechanics in T1DM patients. Regarding disease duration this relationship was not proved.

## Introduction

Type 1 diabetes mellitus (T1DM) is one of the most common chronic disorders affecting young adults. It is associated with serious microvascular and macrovascular complications resulting in at least tenfold increase in cardiovascular diseases as compared with the age matched healthy population^1,2^. Besides, heart failure develops 10–15 years earlier in T1DM patients than in the general population^1,3^. Although in young asymptomatic T1DM patients the standard echocardiographic parameters reflect normal cardiac size and function^4^, tissue Doppler imaging (TDI) and speckle tracking echocardiography seem to be useful techniques for assessing subclinical myocardial involvement in this population. Left ventricular (LV) systolic dysfunction was proved by speckle tracking-derived global longitudinal strain (GLS) data, both in T1DM children and adults^5–12^, whereas TDI measurements suggested impaired LV diastolic function^13–15^. Nevertheless, conflicting results were also reported^5,16–18^. Less is known about right ventricular (RV) function^7,15,19^, or atrial performance^14,20^ in this disease.

Duration of diabetes and quality of the glycemic control have been reported as critical factors contributing toward development of cardiovascular complications^1–3^. Data about their effect on myocardial mechanics, however, are scarce and controversial.

Thus, our work aimed to provide a comprehensive analysis of the myocardial size and function using standard and novel echocardiographic techniques and to investigate the potential associations between disease duration, glycemic control, and the echocardiographic markers of the myocardial mechanics in asymptomatic T1DM patients.

## Methods

### Study population

Seventy-five asymptomatic patients without known cardiovascular disease, diagnosed with T1DM in the tertiary center of 2nd Department of Internal Medicine, University of Pécs were recruited for our prospective study. Detailed medical history was obtained. Patients with impaired LV systolic function (LV ejection fraction (EF) < 55%), significant left-sided valvular disease, atrial fibrillation, known coronary artery disease (CAD), substantial peripheral artery disease or with diabetic nephropathy or retinopathy were excluded from the study. Treadmill exercise test was performed to exclude the patients suspicious for CAD. Blood samples for serum glycated hemoglobin (HbA_1c_%) and other laboratory markers were collected within a 30-day period before inclusion. An age- and gender-matched group of 30 healthy volunteers without any signs or symptoms of cardiac disease was used as control. The study complied with the Declaration of Helsinki. The Regional Research Ethics Committee, Clinical Centre, University of Pécs approved the study. All subjects had given written informed consent before inclusion in the study.

### Echocardiography

Echocardiography was performed using Philips Epiq 7 ultrasound system (Philips Healthcare, Best, The Netherlands) by a single investigator. LV EF was measured by Simpson's method. End-diastolic thickness of the septum and the posterior wall and the end-diastolic diameter of the LV were measured from parasternal long-axis view by M-mode. LV mass was calculated according to the Devereux formula and corrected for body surface area (LVM index)^21^. Relative wall thickness was calculated as 2xposterior wall end-diastolic thickness/LV end-diastolic diameter. LV hypertrophy (LVM index > 115 g in males and > 95 g in females), elevated (≥ 0.43) relative wall thickness and enlarged LV chamber size (LV end-diastolic diameter/height [cm/m] > 3.4 in males and > 3.3 in females) were considered as signs of the hypertensive heart disease^22^.

**Figure 1:**
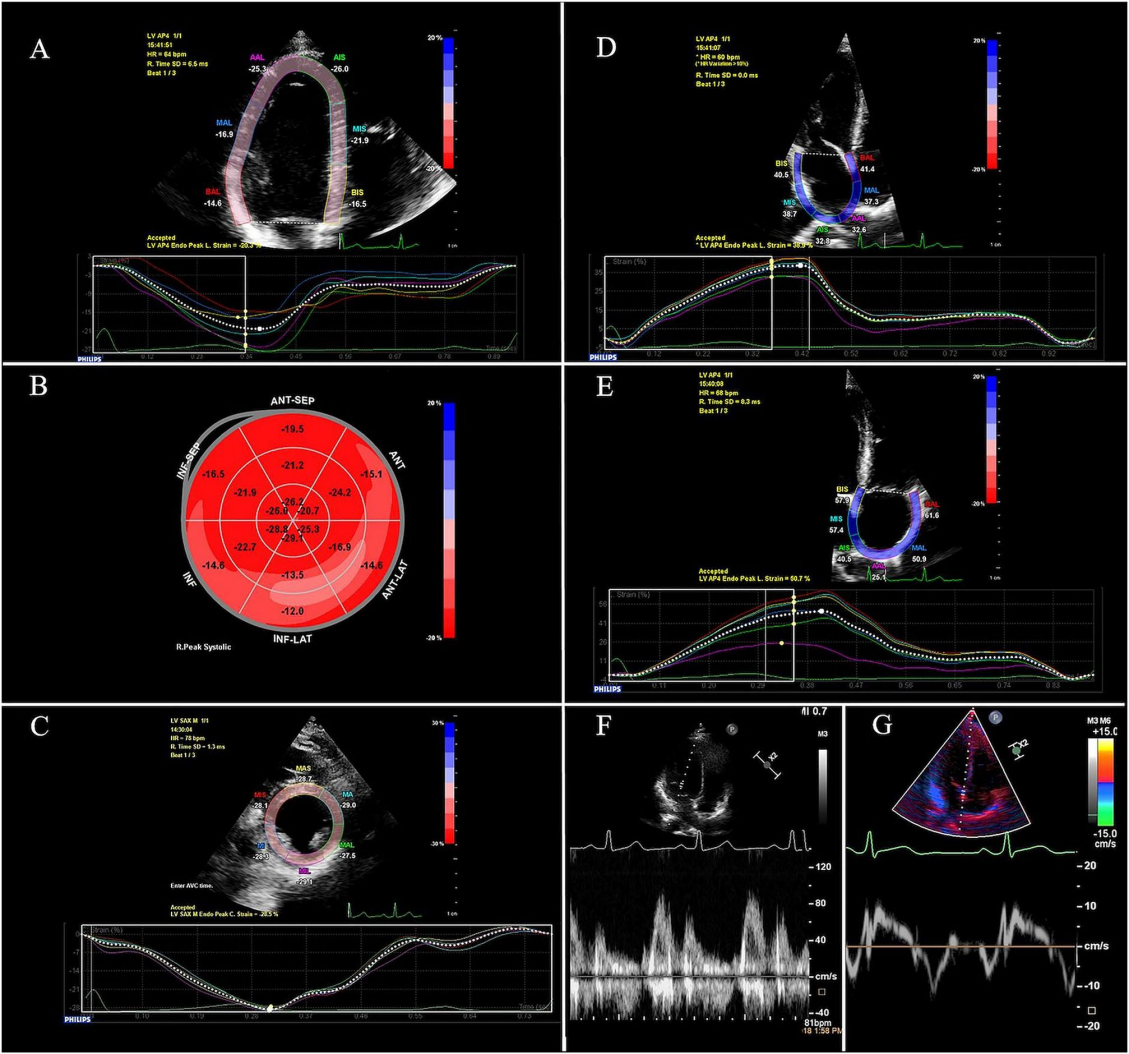
28-year-old male patient, with a 15-year history of T1DM. His HbA1c was 6.5% at inclusion. No signs of hypertensive heart disease. (**A**) Apical four-chamber view depicting the region of interest and the segmental longitudinal strain curves created by the speckle-tracking software. (**B**) Bull’s eye demonstrating the segmental peak systolic longitudinal strain values in the entire LV. Value of GLS is − 20.4%. (**C**) LV parasternal short-axis view at mid-papillary level depicting the region of interest and the segmental circumferential strain curves created by the software. Value of GCS is − 28.5%. (**D**) LA-focused apical four-chamber view depicting the region of interest and the LA strain curve created by the speckle-tracking software. LA reservoir strain: 38.9% (**E**) RA-focused apical four-chamber view depicting the region of interest and the RA strain curve created by the speckle-tracking software. RA reservoir strain: 50.7%. (**F**) Spectral Doppler curve of the mitral inflow. Mitral E: 81 cm/s, Mitral A: 62 cm/s. (**G**) Pulsed tissue Doppler curve measured on the septal border of the mitral annulus. Mitral e′: 11.3 cm/s.

In addition to the spectral Doppler parameters of the transmitral and transtricuspid flow (E, A) Fig. 1F), myocardial systolic (S), early- (e′) and late- (a′) diastolic velocities were measured from apical four-chamber view at the lateral and septal border of the mitral annulus (Fig. 1G), as well as on the lateral border of the tricuspid annulus using pulsed TDI. Lateral and septal mitral annular velocities were averaged. Mitral and tricuspid E/A and E/e′ ratios were calculated^23^. Basal dimension of the RV was obtained at end-diastole in RV-focused apical four-chamber view and corrected for body surface area. As parameters of the RV systolic function, tricuspid annular plane systolic excursion (TAPSE) and RV fractional area change (RVFAC) were measured. Maximal and minimal diameters of the inferior vena cava (IVC) were measured in the subxiphoid view, and collapsibility index (the percent decrease in the diameter of IVC with inspiration) was calculated. RV wall thickness was measured at end-diastole in a zoomed subxiphoid view. Systolic pulmonary artery pressure was estimated as a sum of the pressure difference across the tricuspid valve (calculated using the modified Bernoulli equation) and an estimate of mean right atrial (RA) pressure (5 to 15 mmHg) using the diameter and collapsibility index of the IVC^21,24^. Doppler measurements were obtained from 3 consecutive beats during end-expiratory apnea.

LV diastolic function and filling pressure were classified according to the current recommendation^23^.

### Speckle tracking echocardiography

For strain analysis, three consecutive heart cycles were recorded digitally, and processed off-line, using a dedicated software (QLab, Philips Healthcare, Andover, MA, USA). The analysis was performed by a single investigator, blinded to the clinical and conventional echocardiographic data. In segments with poor tracking, endocardial borders were manually readjusted until better tracking was achieved. To estimate LV GLS, apical four-, three- and two-chamber movies were obtained using 2D echocardiography. Care was taken to obtain true apical images using standard anatomic landmarks in each view. Foreshortening was avoided. The frame rate was set between 50 and 55 frames per second. Regional peak systolic longitudinal strain was determined in all 17 segments from the three apical views (Fig. 1A). The software automatically provided LV GLS as the average of the regional peak systolic longitudinal strain values (Fig. 1B).

Peak systolic circumferential strain was measured from the LV parasternal short-axis view at the mid-papillary level (Fig. 1C). The frame rate was set between 60 and 70 frames per second. Global circumferential strain (GCS) values were calculated as the average of the regional values in the six segments.

For atrial speckle tracking analysis, atrium-focused apical four- and two-chamber view movies (only four-chamber view for RA analysis) were obtained using 2D echocardiography (Fig. 1D,E). The frame rate was set between 80 and 90 frames per second. The onset of the R-wave was set as the reference (zero) point of the strain analysis. The first positive peak of the curve was measured at the end of the reservoir phase, just before the mitral valve opening (reservoir strain). This was followed by a plateau and a second late peak at the onset of the P wave on the electrocardiogram (contractile strain). The conduit strain was defined as the difference between the reservoir and the contractile strain. Results obtained in apical four- and two-chamber views were averaged for left atrial (LA) strain analysis^25^.

Using the atrial borders created for speckle tracking analysis, the same software generated left and right atrial volume curves. Atrial volumes were measured at different time points of the cardiac cycle: maximal volume (Vmax) at the end of T wave on electrocardiogram, just before the opening of the mitral or tricuspid valve; minimal volume (Vmin) at QRS complex, at the closure of the mitral or tricuspid valve; and preceding atrial contraction, at the beginning of P wave (Vp). LA volumes obtained in apical four- and two-chamber views were averaged. All volume values were corrected for body surface area (Vmax-, Vmin-, and Vp index)^26^.

### Statistical analysis

Categorical data were expressed as n (%). The frequencies of categorical variables were compared using chi-square test or Fisher's exact test. The normality of distribution of continuous variables was tested by Shapiro–Wilk test. Continuous variables with normal distribution were presented as mean ± standard deviation; non-normal variables were reported as median (interquartile range). Means of two continuous normally distributed variables were compared by independent samples Student’s t test. Mann–Whitney *U* test was used to compare means of two variables not normally distributed.

Echocardiographic variables that correlate with current HbA_1c_ or disease duration were determined using bivariate Pearson correlation. In a second step, multiple linear regression analysis (enter method) was used. Age-related changes in the echocardiographic parameters are well known, thus age was added to the multiple models. ACE inhibitors/ARBs are often used as treatment or prevention of diabetic nephropathy, regardless of the presence of systemic hypertension. Thus, medical therapy was not useful in identifying patients with hypertension. To eliminate the influence of the hypertension on the echocardiographic variables, signs of hypertensive heart disease were recoded (1 = sign(s) are present; 0 = sign(s) are not present) and used as additional variable in the multiple models. Partial regression plots were used to visualize the correlations between HbA_1c_ and echocardiographic variables (which displays the residuals of each variable after adjusting for age and hypertension). Partial correlation coefficients were reported on the plots.

Patients were subgrouped according to the median HbA_1c_ level and the presence/absence of the hypertensive heart disease. Four groups were created according to the number of cardiovascular risk factors: normal volunteers (NORM); patients below the median HbA_1c_ and without hypertensive heart disease (T1DM-LOW); patients above the median HbA_1c_ but without hypertensive heart disease (T1DM-MED); patients above the median HbA_1c_ and with hypertensive heart disease (T1DM-HIGH). Patients with hypertensive heart disease but below the median HbA_1c_ were excluded from sub-grouping. Comparisons of normally distributed data among multiple groups were performed using one-way ANOVA with LSD post hoc test. Kruskal–Wallis test with Dunn's multiple comparison test was used to compare means of multiple groups of variables not normally distributed.

A p-value of < 0.05 was considered significant. Data were analyzed using IBM SPSS 27 statistical software.

## Results

Of a total of 75 patients, 70 (38.2 ± 11.7 years, 46 female) were eligible for the study. Four subjects were excluded due to inadequate acoustic windows. In one patient, ST changes observed during the exercise test suggested significant CAD, and this diagnosis was later confirmed by coronary angiography. Detailed clinical data of the T1DM patients are reported in Table 1.

**Table 1:** Clinical and echocardiographic data of the T1DM population and comparison with healthy subjects.

	Healthy volunteers (n = 30)	T1DM patients (n = 70)	p
**Clinical characteristics**			
Age (years)	34 (14.25)	38 (20)	0.709
Female gender n (%)	17 (57)	46 (66)	0.390
Body surface area (m^2^)	1.88 ± 0.2	1.84 ± 0.2	0.408
Body mass index (kg/m^2^)	24.8 ± 4.2	23.5 ± 3.6	0.152
Systolic blood pressure (mmHg)	134.0 ± 14.7	135.8 ± 18.1	0.630
Diastolic blood pressure (mmHg)	78.7 ± 8.8	79.8 ± 9.8	0.538
Disease duration (years)		21.0 ± 10.3	
On insulin pump therapy n (%)		47 (67)	
Polyneuropathy n (%)		21 (30)	
**Smoking**			
Never n (%)	22 (73.3)	43 (61.4)	0.505
Previously n (%)	3 (10)	13 (18.6)	
Currently n (%)	5 (16.7)	14 (20)	
**Laboratory data**			
Current HbA1c (%)		7.6 ± 1.3	
Fasting glucose (mmol/l)		8.4 ± 4.6	
Fructosamine (μmol/l)		387.7 ± 65.3	
Creatinine (μmol/l)		73.6 ± 11.7	
eGFR (ml/min/1.73 m^2^)		95.9 ± 19.5	
Hemoglobin (g/l)		138.7 ± 15.2	
Total cholesterol (mmol/l)		4.7 ± 1.2	
Triglyceride (mmol/l)		1.0 ± 0.6	
Erythrocyte sedimentation rate (mm/h)		7.9 ± 7.4	
C-reactive protein (mg/l)		3.1 ± 4.1	
**Medication**			
ACE inhibitors/ARBs n (%)		17 (24)	
Calcium channel blocker n (%)		4 (6)	
Beta receptor antagonists n (%)		12 (17)	
**Echocardiographic characteristics (LV and LA)**			
LV EF (%)	61.1 ± 4.0	62.8 ± 3.2	0.033
LV GLS (%)	− 19.9 ± 2.4	− 19.0 ± 1.9	0.054
LV GCS (%)	− 25.9 ± 3.6	− 28.1 ± 4.7	**0.023**
LVM index (g/m^2^)	80.2 ± 14.4	78.4 ± 16.4	0.611
RWT	0.37 (0.04)	0.40 (0.06)	**0.003**
EDD/height (cm/m)	2.7 (0.3)	2.7 (0.3)	0.117
Mitral E (cm/s)	81.0 ± 11.3	82.5 ± 14.5	0.617
Mitral A (cm/s)	53.8 (13.2)	64.0 (23.4)	**0.008**
Mitral E/A	1.5 ± 0.3	1.4 ± 0.5	0.123
Averaged mitral annular S (cm/s)	10.8 ± 1.4	10.0 ± 1.6	**0.015**
Averaged mitral annular e′ (cm/s)	12.9 ± 1.4	11.1 ± 2.4	** < 0.001**
Averaged mitral annular a′ (cm/s)	9.2 ± 1.9	9.5 ± 1.7	0.525
Mitral E/e′	6.3 ± 0.9	7.8 ± 2.0	** < 0.001**
LA Vmax index (ml/m^2^)	24.6 ± 6.2	25.9 ± 7.8	0.421
LA Vmin index (ml/m^2^)	8.4 (3.3)	8.2 (4.3)	0.513
LA Vp index (ml/m^2^)	13.6 (6.1)	15.0 (6.5)	0.204
LA reservoir strain (%)	35.0 ± 9.5	32.9 ± 7.9	0.272
LA contractile strain (%)	13.8 ± 4.0	13.8 ± 3.9	0.985
LA conduit strain (%)	21.1 ± 7.5	19.1 ± 7.0	0.200
**Echocardiographic characteristics (RV and RA)**			
RVFAC (%)	48.2 ± 8.5	51.2 ± 7.7	0.107
TAPSE (mm)	22.7 ± 2.9	21.7 ± 2.7	0.114
RV wall thickness (mm)	4.5 (1.0)	4.0 (0.5)	0.915
RV basal diameter index (mm/m^2^)	15.0 ± 3.1	15.2 ± 1.6	0.586
PASP (mmHg)	22.9 ± 3.9	23.8 ± 3.8	0.582
Tricuspid E (cm/s)	61.3 ± 10.3	60.8 ± 11.0	0.847
Tricuspid A (cm/s)	40.4 ± 7.1	40.8 ± 9.3	0.836
Tricuspid E/A	1.5 ± 0.3	1.6 ± 0.4	0.138
Tricuspid annular S (cm/s)	13.7 ± 1.7	12.8 ± 2.0	**0.035**
Tricuspid annular e′ (cm/s)	12.6 ± 2.7	11.7 ± 2.8	0.199
Tricuspid annular a′ (cm/s)	11.2 ± 2.7	11.4 ± 3.7	0.747
Tricuspid E/e′	5.1 (1.2)	5.2 (1.6)	0.061
RA Vmax index (ml/m^2^)	21.1 ± 7.4	19.5 ± 5.8	0.298
RA Vmin index (ml/m^2^)	7.8 ± 3.8	7.6 ± 3.2	0.718
RA Vp index (ml/m^2^)	12.8 ± 5.0	12.7 ± 4.5	0.918
RA reservoir strain (%)	50.4 ± 13.9	47.8 ± 12.0	0.356
RA contractile strain (%)	20.5 ± 7.0	20.6 ± 5.7	0.893
RA conduit strain (%)	29.9 ± 11.4	27.2 ± 10.3	0.243

Statistically significant p-values (p < 0.05) are formatted in bold.

### Comparison with healthy subjects

Patients and controls were matched for age, gender, BSA and BMI. LV EF was significantly higher in T1DM patients compared with healthy controls. This difference, however, was clinically not remarkable. More negative GCS values suggested enhanced LV circumferential function in T1DM population. On the contrary, LV GLS values were similar in both groups. Averaged mitral annular S and e′ values were significantly lower, whereas LV E/e′ ratio was significantly higher in T1DM patients, but typically within the normal range. Mitral e′ values suggested impaired relaxation in 11 patients (15.7%). Six (8.6%) of these exhibited normal LV filling pressure, whereas in 5 (7.1%) patients indeterminate LV filling pressure was found.

RWT was significantly higher in T1DM patients. LV hypertrophy, abnormal RWT and elevated EDD/height were found in 6, 23 and 1 cases, respectively (altogether 27 patients).

In the right heart, tricuspid S was significantly reduced in the T1DM population. Real RV systolic dysfunction, however, was rare: RVFAC < 35%, TAPSE < 16 mm and tricuspid S < 10 cm/s were found in 1 (1.4%), 0 (0%) and 3 (4.3%) patients, respectively.

Regarding atrial size or function, no differences were found between the groups. Detailed echocardiographic data of the 70 patients compared to healthy controls are reported in Table 1.

### Correlations between HbA_1c_ or disease duration and the echocardiographic variables

**Figure 2:**
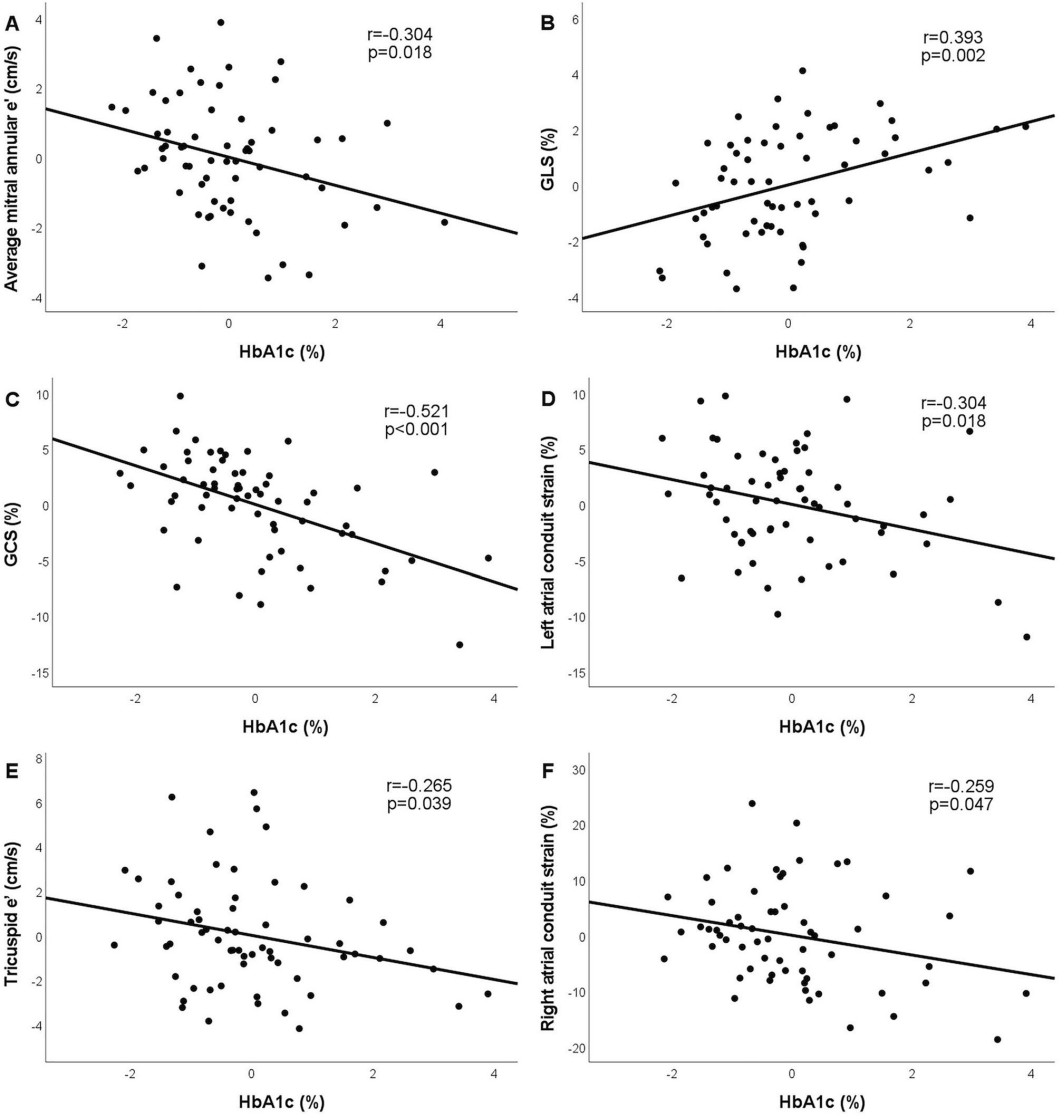
Partial regression plots demonstrate that in age and hypertension adjusted analyses HbA1c (%) correlates with average mitral annular e′ (**A**); LV GLS (**B**); LV GCS (**C**); LA conduit strain (**D**); tricuspid annular e′ (**E**) and with RA conduit strain (**F**). Partial correlation coefficients are reported.

Both HbA_1c_ and disease duration showed significant correlations with various echocardiographic parameters (Table 2). Even when added age and hypertension to the model, current HbA_1c_ level remained independent predictor of LV GLS, LV GCS, mitral and tricuspid e′ and LA and RA conduit strain in multiple linear regression models (Table 3) whereas disease duration lost its significance in similar multiple regression analyses. Partial regression plots demonstrate that HbA_1c_ level correlates significantly with various echocardiographic variables even in age and hypertension adjusted analyses (Fig. 2**)**.

**Table 2:** Significant univariate predictors of the echocardiographic variables in the T1DM population: correlations of current HbA_1c_ and disease duration.

	Correlations of current HbA_1c_ (%)		Correlations of disease duration (years)	
	r	p	r	p
Age (years)	0.186	0.144	0.469	** < 0.001**
LV GLS (%)	0.385	**0.002**	0.076	0.552
LV GCS (%)	− 0.531	** < 0.001**	− 0.127	0.300
Mitral A (cm/s)	0.288	**0.024**	0.087	0.486
Averaged mitral annular S (cm/s)	− 0.221	0.082	− 0.304	**0.012**
Averaged mitral annular e′ (cm/s)	− 0.390	**0.002**	− 0.293	**0.016**
Mitral E/e′	0.329	**0.010**	0.304	**0.014**
LA reservoir strain (%)	− 0.256	**0.045**	− 0.264	**0.031**
LA conduit strain (%)	− 0.353	**0.005**	− 0.312	**0.010**
Tricuspid annular e′ (cm/s)	− 0.330	**0.008**	− 0.227	0.066
RA conduit strain (%)	− 0.326	**0.010**	− 0.212	0.089

Statistically significant p-values (p < 0.05) are formatted in bold.

**Table 3:** Predictors of the echocardiographic variables in T1DM population: multivariate regression analyses.

Variables	B	β	p	F	adj. R^2^	p
**LV GLS (%)**				3.654	0.119	**0.018**
Age (years)	− 0.002	− 0.014	0.910			
Hypertension (0/1)	− 0.486	− 0.124	0.318			
HbA1c (%)	0.568	0.399	**0.002**			
**LV GCS (%)**				22.502	0.268	** < 0.001**
Age (years)	0.025	0.064	0.568			
Hypertension (0/1)	− 1.260	− 0.138	0.216			
HbA1c (%)	− 1.740	− 0.523	** < 0.001**			
**Averaged mitral annular e′ (cm/s)**				22.502	0.514	** < 0.001**
Age (years)	− 0.130	− 0.639	** < 0.001**			
Hypertension (0/1)	− 0.148	− 0.032	0.729			
HbA1c (%)	− 0.403	− 0.227	**0.018**			
**LA conduit strain (%)**				23.224	0.522	** < 0.001**
Age (years)	− 0.392	− 0.664	** < 0.001**			
Hypertension (0/1)	0.308	0.022	0.805			
HbA1c (%)	− 1.115	221	**0.018**			
**Tricuspid annular e′ (cm/s)**				9.035	0.280	** < 0.001**
Age (years)	− 0.112	− 0.448	** < 0.001**			
Hypertension (0/1)	− 0.504	− 0.086	0.431			
HbA1c (%)	− 0.496	− 0.234	**0.039**			
**RA conduit strain (%)**				9.003	0.286	** < 0.001**
Age (years)	− 0.404	− 0.444	** < 0.001**			
Hypertension (0/1)	− 2.869	− 0.132	0.233			
HbA1c (%)	− 1.765	− 0.226	**0.047**			

Unstandardized (B) and standardized (β) regression coefficients. Statistically significant p-values (p < 0.05) are formatted in bold.

### Comparisons between subgroups

**Figure 3:**
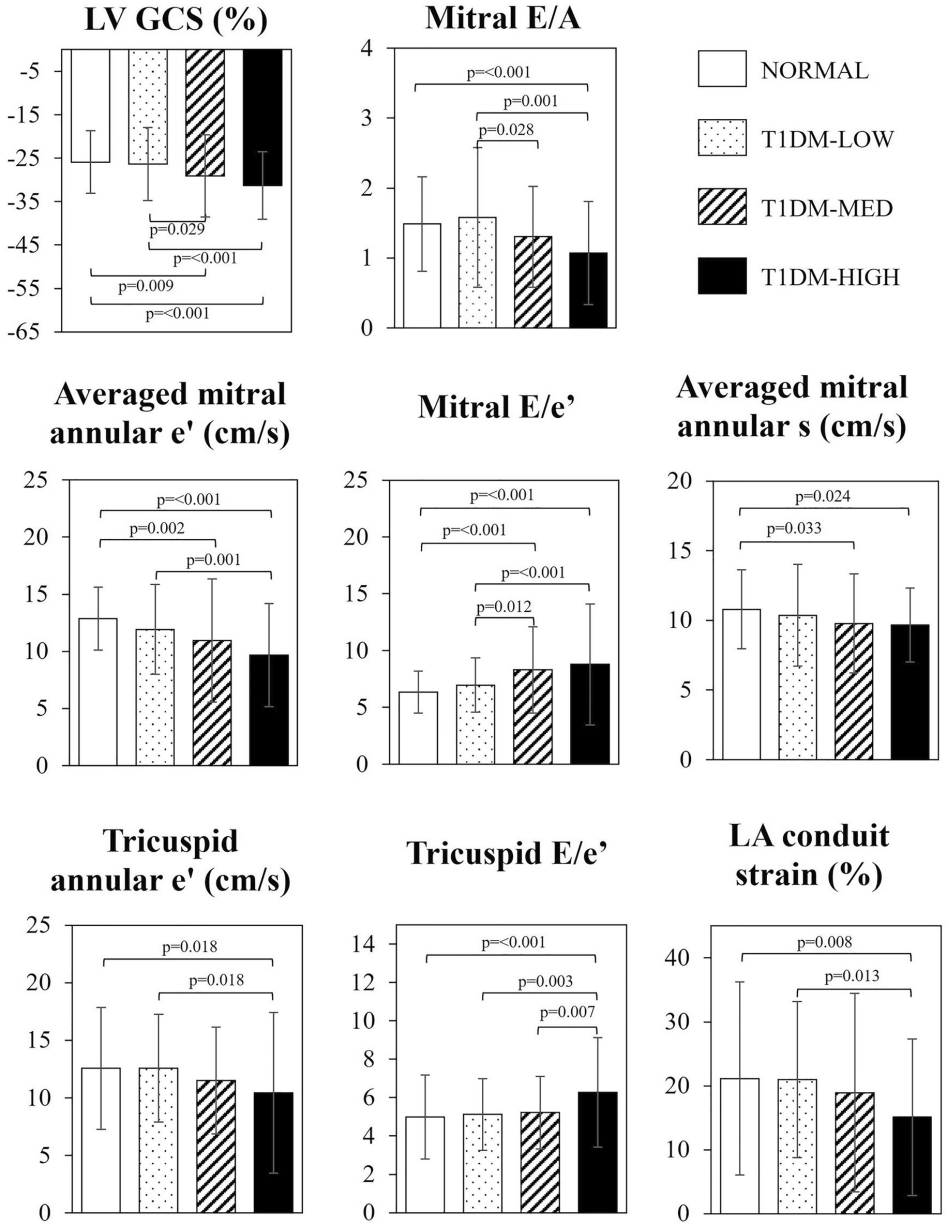
Comparison of echocardiographic variables among healthy subjects and T1DM subgroups.

Median HbA_1c_ level was 7.4 (1.8)% in our T1DM population. Twenty-four patients had HbA_1c_ ≤ 7.4% and no signs of hypertensive heart disease (T1DM-LOW group). Nineteen and sixteen patients exhibited HbA_1c_ > 7.4%, without (T1DM-MED group) or with (T1DM-HIGH group) signs of hypertensive heart disease, respectively. T1DM-HIGH-patients were significantly older than NORM and T1DM-LOW-patients and had significantly higher BMI compared with T1DM-MED and NORM groups. In T1DM-LOW-patients significantly lower LVM index was found compared with NORM. Otherwise, echocardiographic findings in T1DM-LOW-patients did not differ significantly from those in NORM. In T1DM-HIGH-patients LV GLS, mitral S, mitral E/A, mitral e′ and LA conduit strain were significantly reduced, whereas E/e′ values were significantly elevated compared with NORM, reflecting the impairment of LV longitudinal systolic and LV diastolic function. In contrast, LV EF, mitral A and (the absolute value of) GCS were significantly higher in T1DM-HIGH group compared with NORM, suggesting the compensatory behavior of these parameters. Regarding the right heart, tricuspid e′ and RA conduit strain values were significantly reduced, whereas tricuspid E/e′ was significantly elevated in the T1DM-HIGH subgroup. In general, signs of the myocardial involvement were pronounced in T1DM-MED and T1DM-HIGH subgroups compared with T1DM-LOW-patients. The differences between T1DM subgroups, however, were not always significant, especially not between T1DM-MED and T1DM-HIGH subgroups (Fig. 3). Comparison of echocardiographic variables among the study subgroups are reported in Table 4.

**Table 4:** Comparison of echocardiographic variables among healthy subjects and T1DM subgroups.

	Healthy volunteers (n = 30)	T1DM-LOW HbA_1c_ ≤ 7.4% and NO hypertension (n = 24)	T1DM-MED HbA_1c_ > 7.4% and no hypertension (n = 19)	T1DM-HIGH HbA_1c_ > 7.4% and hypertension (n = 16)	p
Age (years)	34 (14.25)	32.5 (17.25)	43 (24)	40.5 (13.75)*^†^	0.097
Female gender n (%)	17 (57)	16 (67)	12 (63)	11 (69)	0.830
Body surface area (m^2^)	1.9 ± 0.2	1.9 ± 0.2	1.8 ± 0.2	1.9 ± 0.2	0.668
Body mass index (kg/m^2^)	23.5 ± 3.6	24.7 ± 3.0	23.9 ± 3.9	27.2 ± 6.1^#¶^	**0.029**
Systolic blood pressure (mmHg)	134.6 ± 14.6	132.5 ± 17.9	129.6 ± 13.4	142.1 ± 15.2^¶^	0.114
Diastolic blood pressure (mmHg)	79.1 ± 8.6	79.3 ± 8.1	77.2 ± 9.8	81.5 ± 10.7	0.582
HbA_1c_ (%)		6.6 ± 0.6^$^	8.7 ± 1.2^§^	8.6 ± 0.8^§^	** < 0.001**
On insulin pump therapy n (%)		19(79)	12 (63)	9 (56)	0.275
LV EF (%)	61.1 ± 4.0	62.5 ± 3.2	61.7 ± 3.4	63.9 ± 2.8*	0.088
LV GLS (%)	− 19.9 ± 2.5	− 19.0 ± 1.7	− 18.7 ± 2.0	− 18.4 ± 2.0*	0.126
LV GCS (%)	− 25.9 ± 3.6^$^	− 26.3 ± 4.2^¶^	− 29.1 ± 4.7^#†^	− 31.3 ± 3.9^#§^	** < 0.001**
LVM index (g/m^2^)	80.2 ± 14.4^†^	71.2 ± 10.3*	75.8 ± 13.0	87.5 ± 20.1^§¶^	**0.005**
RWT	0.38 (0.4)	0.38 (0.4)	0.39 (0.4)	0.45 (0.7)^#§$^	** < 0.001**
EDD/height (cm/m)	2.7 (0.3)	2.7 (0.2)	2.7 (0.3)	2.6 (0.4)	0.157
Mitral E (cm/s)	81.0 ± 11.3	82.1 ± 14.4	87.1 ± 17.1	79.0 ± 11.9	0.332
Mitral A (cm/s)	53.8 (13.2)^$^	53.0 (26.1)^$^	66.9 (19.0)^#§^	74.4 (22.8)^#§^	** < 0.001**
Mitral E/A	1.5 ± 0.3	1.6 ± 0.5^$^	1.3 ± 0.4^†^	1.1 ± 0.4^#§^	** < 0.001**
Averaged mitral annular S (cm/s)	10.8 ± 1.4^⁋^	10.4 ± 1.8	9.8 ± 1.8*	9.7 ± 1.3*	0.067
Averaged mitral annular e′ (cm/s)	12.9 ± 1.4^$^	11.9 ± 2.0	11.0 ± 2.7^#^	9.7 ± 2.3^#§^	** < 0.001**
Averaged mitral annular a′ (cm/s)	9.2 ± 1.9	9.1 ± 1.9	9.4 ± 1.2	10.1 ± 1.6	0.378
Mitral E/e′	6.3 ± 0.9^$^	7.0 ± 1.2^⁋^	8.3 ± 1.9^#†^	8.8 ± 2.7^#§^	** < 0.001**
LA Vmax index (ml/m^2^)	24.6 ± 6.2	25.8 ± 8.2	27.5 ± 8.2	25.8 ± 8.0	0.644
LA Vmin index (ml/m^2^)	8.4 (3.3)	8.1 (4.7)	8.8 (4.8)	8.0 (3.0)	0.560
LA Vp index (ml/m^2^)	13.6 (6.1)	15.0 (6.1)	16.7 (7.4)	15.0 (7.2)	0.453
LA reservoir strain (%)	35.0 ± 9.5	33.9 ± 7.5	32.6 ± 9.5	30.9 ± 5.5	0.468
LA contractile strain (%)	13.8 ± 4.0	12.9 ± 4.0	13.7 ± 3.8	15.8 ± 3.6^†^	0.156
LA conduit strain (%)	21.1 ± 7.5	21.0 ± 6.1	18.9 ± 7.8	15.1 ± 6.1^#†^	**0.039**
RVFAC (%)	48.2 ± 8.5	50.6 ± 9.8	51.8 ± 6.9	51.7 ± 6.6	0.449
TAPSE (mm)	22.7 ± 2.9^†^	21.0 ± 2.0*	22.6 ± 3.0	21.4 ± 2.6	0.075
RV wall thickness (mm)	4.5 (0.8)	4.5 (0.5)	4.0 (0.8)	4.8 (2.0)	**0.242**
RV basal diameter index (mm/m^2^)	15.0 ± 3.1	15.1 ± 1.5	15.8 ± 1.3	14.4 ± 1.4	0.284
PASP (mmHg)	22.9 ± 3.9	24.2 ± 4.3	25.7 ± 4.6	21.5 ± 2.4	0.360
Tricuspid E (cm/s)	61.3 ± 10.3	62.6 ± 9.4	59.5 ± 13.7	59.3 ± 11.0	0.742
Tricuspid A (cm/s)	40.4 ± 7.1	37.8 ± 7.8	42.0 ± 9.4	45.3 ± 10.1^§^	0.060
Tricuspid E/A	1.5 ± 0.3	1.7 ± 0.4^⁋^	1.5 ± 0.4^†^	1.4 ± 0.4^§^	0.050
Tricuspid annular S (cm/s)	13.7 ± 1.7	12.9 ± 1.8	13.3 ± 2.2	12.6 ± 2.2	0.255
Tricuspid annular e′ (cm/s)	12.6 ± 2.7	12.6 ± 2.3	11.5 ± 2.3	10.4 ± 3.5*^†^	0.057
Tricuspid annular a′ (cm/s)	11.2 ± 2.7	10.2 ± 3.3^$^	13.1 ± 4.8^§^	12.1 ± 2.5	0.057
Tricuspid E/e′	5.0 (1.2)	5.1 (1.4)	5.1 (1.4)	6.2 (1.8)^#§$^	**0.005**
RA Vmax index (ml/m^2^)	21.1 ± 7.4	20.0 ± 5.8	19.9 ± 6.9	18.0 ± 5.1	0.563
RA Vmin index (ml/m^2^)	7.8 ± 3.8	7.3 ± 2.6	8.2 ± 4.4	7.2 ± 2.8	0.807
RA Vp index (ml/m^2^)	12.8 ± 5.0	12.2 ± 4.1	13.5 ± 5.6	12.6 ± 4.0	0.856
RA reservoir strain (%)	50.4 ± 13.9	49.5 ± 8.9	46.9 ± 15.4	45.4 ± 14.4	0.629
RA contractile strain (%)	20.5 ± 7.0	17.9 ± 4.7^¶^	21.6 ± 4.7^†^	23.0 ± 5.2^†^	0.051
RA conduit strain (%)	29.9 ± 11.4	31.6 ± 8.3	25.3 ± 12.8	22.4 ± 10.3*^†^	**0.048**

Statistically significant p-values (p < 0.05) are formatted in bold. *p < 0.05 versus NORM; ^#^p < 0.01 versus NORM; ^†^p < 0.05 versus T1DM-LOW; ^§^p < 0.01 versus T1DM-LOW; ^¶^p < 0.05 versus T1DM-MED; ^$^p < 0.01 versus T1DM-MED.

## Discussion

Severe micro-, and macrovascular complications are common in patients with advanced T1DM. Subclinical myocardial involvement, however, may be present even in T1DM patients without manifest heart disease. Conventional echocardiographic techniques may fail, but TDI and speckle tracking echocardiography seem to be useful for recognizing subclinical myocardial involvement in this population. Disease duration and quality of the glycemic control are known as the most important factors responsible for the development of cardiovascular complications in T1DM^1–3^. Significant correlations have already been reported between disease duration, glycemic control, and the novel parameters of the myocardial mechanics^5,8,15,18,20^. Literature data, however, are contradictory regarding these questions. Therefore, we aimed to explore the size and mechanics of all cardiac chambers in asymptomatic T1DM patients. Potential associations between disease duration, glycemic control, and the echocardiographic parameters were also investigated. We tried to avoid the confounding effect of the age and hypertensive heart disease.

Similarly to the previous findings^4^, LV EF was preserved, LV size was normal in T1DM patients compared to the normal population. LVM index was enlarged only in patients with hypertensive heart disease. On the other hand, in T1DM patients with well controlled diabetes and without hypertension, LVM index was significantly lower than in healthy persons. This result is in line with the previous cardiac CT findings obtained in normoalbuminuric T1DM patients^27^. Similarly, Aepfelbacher et al. reported that improved glycemic control induces regression of LVM in patients with T1DM^28^. Better glycemic control may reduce LVM by improving intracellular calcium handling and reducing myocyte calcium overload in this population^29^. Another potential mechanism is the insulin-mediated reduction in elevated growth hormone levels parallel with the improved glycemic control, as elevated growth hormone levels are associated with LV hypertrophy in diabetes mellitus^30^.

Although Karamitsos et al. reported preserved LV longitudinal systolic function in T1DM patients^15^, average mitral annular S value was significantly reduced in in our patients. Similarly to their results, mitral annular S showed significant correlation with disease duration. After correcting with confounding factors, however, this significance was lost in our study.

**Figure 4:**
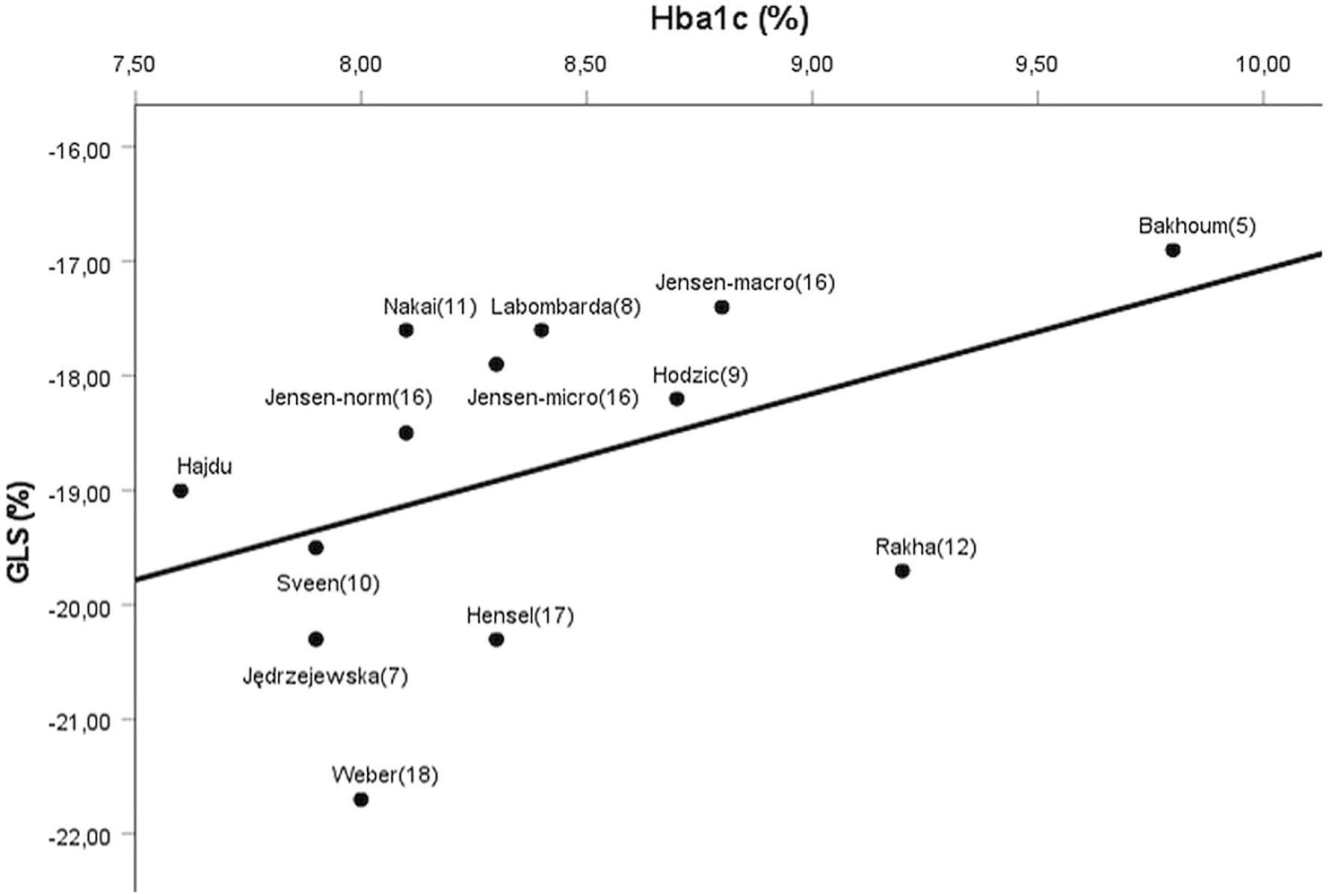
Results of the previous studies suggest a linear relationship between HbA_1c_ (%) and LV GLS (%) at global level. Good glycemic control is associated with preserved, whereas worse glycemic control is associated with impaired LV GLS. Confounding factors (e.g. age) and inter-vendor variability of the GLS were neglected. [Studies are referred by the name of the first author and by the reference number. Jensen-norm: patients without albuminuria (16), Jensen-micro: patients with microalbuminuria (16), Jensen-macro: patients with macroalbuminuria (16)].

LV GLS demonstrated subclinically impaired LV systolic function in multiple studies, not only in T1DM patients with known CAD^31^, but even in T1DM children and adults without the evidence of cardiovascular disease^5,7–12^. On the other hand, Jansen et al. reported preserved LV GLS values in T1DM patients without albuminuria (representing a relatively low cardiovascular risk profile/good glycemic control)^16^. Besides, in the studies of Hensel et al.^17^ and Weber et al.^18^, relatively good glycemic control was associated with preserved LV GLS. Similarly to the latter findings, mean LV GLS value was preserved in our T1DM population, compared with healthy controls. In patients with HbA_1c_ above the median, however, significantly impaired LV GLS values were found. Bakhoum et al. found that HbA_1c_, but not the disease duration, was independent predictor of GLS^5^. Similar findings were reported by Labombarda et al.^8^. In our study, HbA_1c_ levels showed significant relation with LV GLS values, even in age and hypertension adjusted multiple regression model. By reviewing the results of the previous studies this tendency is confirmed even at global level: in T1DM populations with relatively good glycemic control the mean LV GLS is preserved^16–18^, whereas worse glycemic control is associated with impaired GLS^5,7–12^ (Fig. 4).

Data regarding LV GCS are contradictory in the literature. In a pediatric T1DM population Rakha et al. reported reduced GCS values compared with controls^12^. In the study of Jędrzejewska et al., GCS was reduced in the basal segments, but preserved at mid-papillary and apical levels^7^. Preserved GCS values were reported in many T1DM populations, compared with healthy subjects^5,8,17^. In contrast to the previous results, elevated GCS values were revealed in our study, even at population level, but especially in patients with HbA_1c_ above the mean. Our finding is not unique in the literature: According to the current theory, impaired LV longitudinal function is compensated by enhanced circumferential function, thus LV EF remains preserved. The shift from longitudinal to circumferential shortening was first recognized in hypertensive patients^32^, but it has also been reported in T1DM patients^33^. Significant associations between LV GCS and HbA_1c_ or disease duration have never been reported before. In our study, however, HbA_1c_ remained significant independent predictor of this parameter in multiple regression model.

Although both reduced mitral annular S and elevated LV GCS values reflect impaired LV longitudinal systolic function, LV GLS remained preserved in our T1DM population (with a borderline p value). These data may suggest that LV GLS is less sensitive to the mild changes in LV longitudinal systolic function than the other two parameters.

Literature data about LV diastolic function are inconsistent in T1DM patients. Di Cori et al. did not detect any diastolic functional abnormalities in young asymptomatic patients with TDM1^34^. Prevalence of diastolic dysfunction was reported as 8% by Palmieri et al.^4^, whereas 27.4% of the patients had diastolic damage in the work of Raev et al.^35^. Discrepancies between these results may be explained not only by the variability of the populations but by the various echocardiographic methods applied in these studies. Regarding the modern, tissue Doppler-derived parameters, the result are similarly contradictory. Bakhum et al. reported, that mitral annular e′ values were preserved, but E/e′ values were higher in T1DM patients than those in healthy subjects^5^. In contrast, Weber et al. detected mildly reduced mitral e′ values. Mitral E/e′, however, was not elevated in their study^18^. Finally, in the studies of Jędrzejewska et al. and Gul et al., mitral annular e′ values were significantly lower, whereas LV E/e′ ratio was significantly higher in T1DM patients compared with healthy subjects, but typically within the normal range^7,13^. Our findings are in line with the latter studies: in our study-population, mitral annular e′ values were reduced, whereas mitral E/e′ values were elevated, but mainly within the normal range. LV diastolic dysfunction was found in 15.7% of our patients.

Neither HbA_1c_ nor disease duration showed significant correlation with the parameters of LV diastolic function in the study of Bakhoum et al.^5^. In contrast, mitral annular e′ correlated significantly both with HbA_1c_ values and disease duration, as reported by Weber et al.^18^. Although tissue Doppler parameters were not used in their study, Grandi et al. reported that diastolic dysfunction could be prevented and reversed by tight glycemic control in T1DM patients^36^. Our results are partially in agreement with the latter studies: both mitral annular e′ and E/e′ showed significant correlation both with HbA_1c_ and disease duration in our population. In multiple regression model, however, only the association between HbA_1c_ and mitral e′ remained significant.

Similarly to the left heart, Karamitsos et al. revealed preserved RV longitudinal systolic function in T1DM, whereas tricuspid e′ was reduced in their patients, suggesting impaired RV relaxation^15^. In contrast, in the study of Ahmed et al., a young T1DM group showed statistically significant decrease in tricuspid S and in RV longitudinal strain. Tricuspid E/e’ was significantly elevated, but tricuspid e′ was preserved in their study^19^. Although TDI-derived parameters of their RV function were preserved, Jȩdrzejewska et al. also reported significantly impaired RV longitudinal strain in T1DM patients^7^. At population level, tricuspid S was significantly reduced in our study, but no significant reduction of tricuspid e′ was proved. On the other hand, in the subgroup of T1DM patients with less well controlled diabetes and hypertensive heart disease, tricuspid e′ was significantly reduced, whereas tricuspid E/e′ was significantly elevated, suggesting RV diastolic dysfunction. Correlations between RV function and HbA_1c_ or disease duration have not been reported in the literature. In our population tricuspid e′ showed significant association with HbA_1c_ level, even in multiple regression model.

Nemes et al. reported enlarged LA volumes in T1DM patients^37^. This finding, however, was not confirmed in our study. Acar et al. investigated LA function based on volumetric measurements, in T1DM children and adolescents. LA passive emptying fraction, reflecting LA conduit function, was decreased, whereas LA active emptying fraction, reflecting contractile function, was increased in their patients compared with healthy controls^14^. Similarly, Ifuku et al. reported reduced LA reservoir and conduit strain in adolescents and young adults with T1DM^20^. Regarding RA strain, data are available only in T2DM patients: Tadic et al. reported reduced reservoir and conduit but enhanced contractile strain both in LA and RA^38^. In contrast, at population level, both LA and RA phasic functions were found to be preserved in our T1DM patients compared with healthy controls. Nevertheless, in the subgroup of patients with less well controlled diabetes and hypertensive heart disease, both LA and RA conduit strain were significantly reduced compared with healthy subjects, whereas compensatory increase of LA and RA contractile strain were found when compared with those having well controlled diabetes. This compensatory behavior of LA contractile function—parallel with the decline of LV diastolic function—has already been reported in other conditions^39,40^. LA conduit strain showed significant correlation with disease duration, as reported by Ifuku et al.^20^. Besides, in T2DM, LA conduit and reservoir strain, as well as RA conduit and contractile strain were independently associated with HbA_1c_ levels^38^. In our T1DM population, even in multiple regression model, HbA1c levels showed significant associations with LA and RA conduit strain. Significant correlations were also proved between LA reservoir and conduit strain and disease duration. In multiple regression models, however, the latter associations were lost.

Regarding myocardial mechanics, our results are only partially in agreement with the previous findings. Nevertheless, due to the lack of uniformity in patient-selection (regarding age, disease duration, glycemic control, comorbidities) and in the use of the echocardiographic techniques, the results of the studies are not easy to compare. Based on their mean HbA_1c_ level, our asymptomatic T1DM population exhibited a relatively well controlled diabetes compared with the populations reported in the previous studies. This may, at least partially, explain the differences.

As new finding, our comprehensive analysis suggests that in asymptomatic T1DM patients, glycemic control has significant impact on the mechanics of all cardiac chambers, even after careful correction for the main confounding factors. This early myocardial damage forms the basis of the future heart failure in patients with poor glycemic control. Our results are in line with the large observational study performed in a cohort of 20,985 T1DM patients by Lind et al. A strong positive association was found between HbA_1c_ and the risk of heart failure in their study^3^.

Numerous limitations of our study need to be acknowledged. First, our study population was limited in size due to the challenging selection of patients without cardiovascular comorbidities. Neither daily physical activity, nor statin or fibrate use of the patients are reported. Treadmill exercise test was performed to exclude patients suspicious for CAD. Due to ethical reasons, use of coronary angiography was avoided in asymptomatic subjects.

Patients with well-controlled hypertension were not excluded from the study, but we intended to eliminate the confounding effect of hypertension in our analysis.

For obtaining atrial strain values, we used a software that was developed for LV strain analysis because dedicated software was not available. RV strain may better reflect the subclinical impairment of the RV systolic function than our traditional and tissue Doppler parameters. Nevertheless, in the lack of appropriate analytical software, RV strain analysis was not performed in our study.

Alpha level was not adjusted when multiple comparisons were made. This may increase the chance of type 1 error (false positives).

## Conclusion

Our data suggest that quality of the glycemic control has a significant impact on the subclinical myocardial involvement in T1DM patients. Regarding disease duration, we could not prove this relationship. Thus, tight glycemic control must be a high-priority therapeutic aim for diabetic patients to minimize the risk of myocardial damage and consequential heart failure.

## Data Availability

The datasets used and analyzed during the current study are available from the corresponding author on reasonable request.
